# Understanding the Current Health Services Research Workforce and Maximizing its Future

**DOI:** 10.1111/1475-6773.13038

**Published:** 2018-09-21

**Authors:** Meghan J. Wolfe, Lisa A. Simpson, Nir Menachemi

**Affiliations:** ^1^ Department of Health Policy and Management Milken Institute School of Public Health The George Washington University Washington DC; ^2^ AcademyHealth Washington DC; ^3^ Richard M. Fairbanks School of Public Health Department of Health Policy and Management Indiana University Indianapolis IN

**Keywords:** Workforce, training programs, funding

## Abstract

In 2016, AcademyHealth continued its longstanding efforts to understand the health services research (HSR) workforce, to inform its changing needs through the commissioning of several papers and an invitational conference. This paper serves to summarize the commissioned studies that appear in the current issue of this journal.

Defining the evolving boundaries of health services research and its workforce remains a challenging and complex exercise. As a multidisciplinary field, health service research has, at times, struggled to remain broad enough to encompass the diverse interests of its members while trying to become more precise for the purposes of branding and advocacy work. This tension has created obstacles for understanding the current stock of the health services research workforce, as well as its future needs and direction. In the context of this background, AcademyHealth hosted a strategic conference of key stakeholders of the health services research workforce to plan for the future of the field. A description of the planning process and resulting recommendations from the conference is available in the current issue (Menachemi, Wolfe, and Simpson 2018). In addition, AcademyHealth commissioned a variety of analyses that were shared with conference participants and appear as full manuscripts in the current supplement issue of the journal. Below is a description of each of these featured papers which collectively seek to address many of the above challenges, as well as some opportunities for the field of health services research to advance training programs, increase diversity, and ultimately produce higher quality, more impactful research.

## Overview of Featured Papers

The featured papers examine (1) the current stock of health services researchers (Frogner 2018); (2) employment demand for health services researchers (Rich and Collins 2018); (3) trends in health services research funding (Simpson et al. 2018); (4) funding trends for the training of health services researchers (Mor and Wallace 2018); (4) issues and trends in the global health policy and systems research workforce (Javadi, Tran, and Ghaffar 2018); (6) updates to the health services research doctoral core competencies (Burgess, Menachemi, and Maciejewski 2018); (7) updates to the Canadian health services and policy research core competencies (Bornstein et al. 2018); and (8) recommendations for supporting the growth and evolution of the health services research workforce.

One of the primary issues within the field of health services research is identifying its bounds. Frogner's work seeks to estimate the number of health services researchers and the industries within which they work. By leveraging numerous data sources, including new social media sources, and building on the work of McGinnis and Moore (2009), Frogner is able to provide an updated estimate for the number of individuals who identify as health services researchers.

In the current supplement issue, authors examine the ways in which the field of health services research measures and understands the pipeline of new researchers into the field. Because the field of health services research is inherently interdisciplinary, students entering the field do not just come from traditional health services research doctoral programs. Researchers in the field hold advanced degrees in public health, medicine, law, and other social and basic sciences, making it difficult to predict how the field of health services research will grow and evolve. Frogner looks more closely at health services research‐related training programs and provides guidance on how the field might change and grow in the foreseeable future.

Whereas Frogner examines the current stock of health services researchers, as well as future supply of students entering the field, Rich and Collins examine the current and future demand for health services researchers. Through interviews with leaders working in diverse sectors that employ health services researchers, Rich and Collins were able to characterize the current and future needs of employers in the field and identify skills gaps that training programs in the field might address. Many common themes emerged from the interviews despite the informants representing different settings and a focus on different topics of health services research.

The set of papers from Simpson and colleagues, and from Mor and Wallace provide an overview of current federal funding trends in health services research and funding for health services research training programs, respectively. From these it is clear that the vast majority of public funding for health services research comes from the National Institutes of Health and that recent pressures on federal discretionary spending as well as enduring questions about the value of funding health services research have slowed growth in funding.

In examining funding for training programs, Mor and Wallace provide an overview on the current funding climate and explore alternative funding and training models that health services research training programs may consider adopting to address some of the skills gaps raised by Rich and Collins. In particular, they discuss the importance of training in applied setting and in diverse fields and sectors to provide new researchers with the pragmatic and business‐minded experience that employers seek. Mor and Wallace's commentary should serve as a starting point for health services research training programs that are looking to innovate and grow during a time where funding is more uncertain and potentially more precarious.

Javadi and colleagues provide a global perspective on the health policy and systems research workforce, its relationship to health services research, and its importance to achieving the Sustainable Development Goals adopted by countries in 2015. Outlining similar challenges to those faced by the US‐based workforce, the authors reflect on challenges and strategies in managing the global health policy and systems research workforce in order to stimulate dialogue and learning across similar fields.

Burgess and colleagues make a significant contribution to health services research training programs in their commentary, which provides an update of the doctoral core competencies to reflect advances in methods and topic‐specific proficiencies that have emerged over the past several years. While a number of these remain unchanged from the previous version (Forrest et al. 2009), they also speak to the many new developments in the field. Much like Burgess et al., Brown and colleagues worked with various stakeholders in the health services and policy research field, including leaders of training programs, health policy and health services practitioners, and funders, to update the core competencies for doctoral HSPR trainees in Canada. Drawing many parallel conclusions, the authors of both papers propose changes to the doctoral competencies that emphasize applied skillsets and equip trainees with a range of proficiencies, including analyzing complex problems using a variety of methods, interdisciplinary work skills, and knowledge translation.

The final paper in the current supplement issues outlines a set of recommendations that emerged from a consensus‐building process of a group of stakeholders that was facilitated by AcademyHealth. Menachemi and colleagues share this process and the resulting action areas that AcademyHealth will engage in to continue to monitor and report on the status of the health services research workforce.

## Summary

The topics and trends described in this paper are strikingly similar to conclusions drawn by fields related to health services research, such as the biomedical workforce and the clinical workforce (Zerhouni et al. 2016; Meggeness et al. 2017). These fields are also experiencing radical shifts including the expansion of types of employers, changes in training and career trajectories, and a diversifying workforce.

In April 2018, the National Academies of Science, Engineering and Medicine (NASEM) released a report on the biomedical research workforce, including health services research (NASEM 2018). It documents the findings of earlier reports and includes a number of recommendations which align with the recommendations put forth from the AcademyHealth Workforce Initiative Task Force that are outlined in this supplement. For example, the report notes the continued decrease in the number of Ph.D.'s who secure tenure‐track positions and the increasing average age at which an R01 is secured, both indicators of the growing difficulty of establishing independent research careers in academic settings. The committee also notes the mismatch between the training that is received and the available career opportunities outside of academic settings, a key finding of Collins and Rich. The NASEM report goes on to recommend that “Congress and the National Institutes of Health (NIH) should create and expand existing entrepreneurial and private‐sector opportunities”, echoing the recommendations in this supplement. In another example, the report calls for more transparency and accountability for monitoring the research workforce, including its inclusion of underrepresented racial/ethnic minorities, another recommendation consistent with those for health services research.

The papers in the current issue provide a snapshot of the challenges and opportunities facing the health services research workforce, and fundamentally, our field. Some of these are not new and must continued to be grappled with, such as identifying the bounds of the field and supporting a diverse pipeline of researchers, while others signal shifts in the realities of funding availability and responses to the changing needs of employers. The environment is different due in part to the explosion of new data, a stronger focus on social determinants and population health, far greater demand for stakeholder engagement, and renewed – and perhaps louder – calls for reducing the costs and increasing the quality of healthcare. With these and other changes in the overall health care and research ecosystems, AcademyHealth and the field must continue to ensure that important research questions are being addressed so that evidence can inform policy and practice changes designed to ultimately improve health and health care.

## Supporting information

Appendix SA1: Author Matrix.
